# The experiences of sex workers accessing HIV care services in Bulawayo, Zimbabwe

**DOI:** 10.4314/ahs.v21i2.14

**Published:** 2021-06

**Authors:** Idah Moyo, Margaret Macherera

**Affiliations:** 1 HIV Services Department. Population Services International-Zimbabwe, Harare, Zimbabwe; 2 Department Environmental Science and Health, Faculty of Applied Science, National University of Science and Technology, Bulawayo, Zimbabwe

**Keywords:** Linkage to care, retention in care, enabling environment, facilitators, barriers

## Abstract

**Background:**

Although sub-Saharan African countries have rolled out massive HIV treatment and care programmes, there is little evidence of these having embraced key population groups particularly female sex workers. Due to the criminalisation of sex work in countries like Zimbabwe, research on HIV and its impact on this group is sparse. The absence of an enabling environment has hindered access to HIV care and treatment services for female sex workers.

**Objectives:**

To gain an in-depth understanding of the experiences of female sex workers accessing HIV care and treatment services to enhance programming and planning for this key population group.

**Methods:**

This study was qualitative and phenomenological. Data saturation determined the sample size of 20 participants. Data was collected using in-depth interviews that were audio recorded, transcribed, and subjected to thematic content analysis.

**Results:**

Our findings demonstrate varying dynamics between the private and public sector HIV care services for sex workers, with facilitators and barriers to access to care.

**Conclusion:**

Health workers need sensitization and training in the provision of differentiated care. For effective linkage to and retention in care an enabling environment is critical.

## Introduction

Key populations are disproportionately affected by HIV despite constituting a small proportion of the general population globally^1^. In the context of Zimbabwe, it is estimated that the size estimate for female sex workers is 127, 385^2^ versus a population of 4, 327 812^3^ females aged 15 years and above. Statistics indicate that in 2018, globally, 54% of the new infections were from key populations and their partners^1^. The World Health Organization (WHO)^4^ defines key populations as populations who are at higher risk for HIV irrespective of the epidemic type or local context and who face social and legal challenges that increase their vulnerability. They include sex workers, men who have sex with men, transgender people, people who inject drugs, and people in prison and other closed settings. According to WHO^4^ sex workers include female, male and transgender adults (18 years of age and above) who receive money or goods in exchange for sexual services that are consensual between adults. WHO further notes that due to legal and social constraints, these populations have not been prioritised in HIV programming. To achieve HIV epidemic control, it is critical that engagement of female sex workers(FSW) (who are relatively more vulnerable than their female counterparts in the general population)^4^ for HIV care and treatment services is strengthened. According to e UNAIDS, the risk of acquiring HIV in 2018 was 21 times higher for sex workers than adults aged 15–49 years and this group accounted for 6% of new infections globally^1^. Despite being the most affected by HIV, key population groups still have reduced access to HIV care. They face barriers to treatment associated with stigma and discrimination and criminalization of their services and behaviour making it difficult to track various steps along the HIV care continuum^1^,^5^,^6^,^7^.

HIV epidemiology, access, and utilization of HIV care services among FSW in sub-Saharan Africa remain poorly understood. In 2018, 25% of the new infections in eastern and southern Africa were from key populations and their partners (3% from sex workers, whilst 10% from clients of sex workers and sex partner of other key populations, 12% from other key population goups). UNAIDS estimated that 37,9 million people were living with HIV and there were 1.7 million new infections globally at the end of 2018. Sub-Saharan Africa is still at the epicentre of the global HIV epidemic, with 61% of all HIV new infections occurring in this region in 2018^1^. Bulawayo, in Zimbabwe where this study was undertaken, had a population of 676, 650^8^. The three Matabeleland provinces (among which Bulawayo falls) have the highest HIV prevalence in Zimbabwe, disaggregated thus: Matabeleland North (18.3%), Matabeleland South (21.2%) and Bulawayo Metropolitan (19.1%)^9^.

A cohort analysis of data for sex workers in Zimbabwe from 2009 to 2013 showed an HIV incidence of more than 10 new cases per 100 person-years at risk^10^. A population-based survey from the Sisters Antiretroviral therapy Programme for Prevention of HIV, showed that in Zimbabwe the HIV prevalence among sex workers was 57.1% in 2018^11^. In eastern and southern Africa, for the majority of the countries, epidemiological data on key populations is either scanty or non-existent^1^,^12^. Effective programming targeting this group depends on evidence-based research. In line with the 95,95,95 UNAIDS strategy and commitments that aim at accelerating HIV response and a reduction in new HIV infections, it is critical that key population groups have access to HIV care services. WHO posits that it is difficult to estimate the full extent of key population access to services due to a lack of data and challenges in data disaggregation^13^. Curbing the HIV epidemic will not be possible without reaching key populations such as sex workers who carry a higher risk of HIV infection and have a poorer access to HIV prevention and care^13^,^14^,^15^. Therefore, it was important to gain an in-depth understanding of the lived experiences of this group as they accessed HIV care and treatment services in Bulawayo, since they are vulnerable to the epidemic. In line with strategies of achieving HIV epidemic control, the findings of this study are critical in programming for key populations.

## Methods

### Study design

A phenomenological qualitative descriptive study was conducted. It explored and described the lived experiences of HIV positive FSW who accessed HIV care services.

### Study setting

The study was set in state owned and private health care facilities in Bulawayo, Zimbabwe that offer HIV care services (HIV testing, ART initiation, adherence counselling and viral load monitoring). The service providers in these facilities included: nurses, primary care counsellors and the medical doctors. The operating hours varied in these clinics; with the state-owned facilities opening from 8am to 4pm from Monday to Friday and 8am to 1pm on Saturday, whilst the private clinics were open on Monday to Saturday from 8am to 4pm. Generally, the state-owned facilities cater for all population groups and are high volume facilities. On the other hand, the private ones largely focus on increasing service access to key population groups. A total of five facilities were included. Of these, one was private while the other four were state owned. This information was established during the interview process since the participants were recruited from a key population organisation and not from the health facilities.

### Study Population

The study population was of HIV positive female sex workers that were utilising HIV care services in public and private health care facilities in Bulawayo. In this study, the term sex worker was used to refer to any female aged 18 years and above who consents to having sex with a man for monetary or other benefits as an occupation. Whilst young women selling sex (below the age of 18 years) could have been available at the study setting, they were not included as study participants for ethical reasons since parental consent would have been required. This may have been difficult since parents/guardians may not be aware of their occupation as sex workers. Admittedly, their inclusion could have brought different and unique experiences in accessing HIV care and treatment services. Judgemental purposive sampling was used to recruit participants for this study^16^. The inclusion criteria for participating in the study was as follows: being a self-identified, female sex worker living with HIV, aged 18years and above, receiving HIV care services in the study settings and been on antiretroviral therapy for a period of 6 months to 5 years(a period perceived to be long enough for the participants to have gained experiences relevant for the phenomenon under study). Participants were invited by telephone to take part in the study after their contact details were obtained from an organization that provides services for key population groups. The sample size of 20 participants was determined by data saturation. For this study, saturation was attained (at participant number fifteen) when no new information emerged and there was only repetition of previously collected data. Five more additional participants were interviewed before closure was reached at participant number 20.

### Data Collection

Data was collected from 15 December 2018 to 15 March 2019. All study participants were interviewed in Ndebele by two researchers who had the competence and experience of working with sex workers. The interviews were unstructured, in-depth, and open-ended. The researchers initially posed a grand tour question as follows: “What have been your experiences as you accessed care in the clinics from the time you tested HIV positive up to now?” Probing questions were further asked to ensure clarity. To ensure privacy and confidentiality, the research participants were always interviewed in a selected private room. To ensure authenticity and a clear audit trail, the interviews were audio recorded and later transcribed verbatim by both researchers within 48 hours of conducting the interview. The researchers worked on the transcriptions independently and later compared notes. Since both researchers are fluent in both languages, they translated the transcriptions from Ndebele to English. An independent analyst was requested to analyse all the transcripts and this report was compared with initial results for data analysis as a measure to enhance credibility of data. The interpretations were discussed and verified with the researchers. Both researchers are public health specialists with vast experience in qualitative research. One of them teaches public health at a local university, while the other works for a Non-Governmental Organization that provides services for key populations.

The researchers also made use of field notes during data collection. A reflexive diary was utilised as an organisational tool in general. It was also used for capturing non-verbal expressions of the interviewee and noting anomalies during the interview. The same diary was also used to reflect on the interview experience, capture the dominant themes and how the researchers felt during the interviews. The duration of each interview was about 30 to 45 minutes for all participants to express their views regarding the phenomenon under study.

### Measures to ensure trustworthiness

To ensure trustworthiness and rigour, the following measures were taken: use of reflexive diary, co-coder, peer debriefing, keeping a detailed records and member checking^17^. A reflective diary was kept by researchers for ensuring neutrality and objectivity. The reflexive diary was used to among other things capture the emotional state of the researcher such as ‘feeling angry’ which could negatively impact on the researcher's objectivity. Peer evaluation and use of a third person (co-coder) to code data with the researchers were used to enhance credibility and inter-coder reliability was established. Records were kept locked in a cupboard in the office of the principal investigator. This way an audit trail was left to be followed by any researcher. To enhance honest and deeper reflective analysis by the researchers, peer debriefing was done, and irregularities identified were addressed. By coding and recoding comparing the themes and categories with a co-coder, researchers ensured dependability. To enhance authenticity, verbatim extracts from the interviews were utilised. To further enhance credibility, member checking was used when the researchers went over the individual transcripts with each participant. A week after the interview, the researchers re-engaged and invited participants through telephone calls to the same venue of the initial interview to review their individual transcripts. This was meant to give the research participants an opportunity to react, to ensure that there were no distortions or missing data and to allow them to check and verify information recorded^17^. No departures were noted from what they had said.

### Data Analysis

For sample description, socio-demographic data were summarized. The two researchers transcribed the data verbatim. Transcripts were then analysed manually using thematic analysis according to Braun and Clark^18^,^19^. Both researchers, a co-coder and an independent analyst were involved in data analysis.

### Ethical Measures

The research study had ethical approval from the Medical Research Council of Zimbabwe [MRCZ] (Ethics Clearance Number MRCZ/A/2338) and Ministry of Health and Child Welfare. Authority to gain access to the study participants was obtained from the Sexual Rights Centre. Written informed consent was obtained according to principles of good clinical practice. The purpose, benefits and possible risks were explained to study participants. Also, the fact that participation was purely voluntary and that the interviews were going to be audio recorded was highlighted. The research participants were also informed that they had a right to withdraw from the study at any time if they so wished, without even giving the reason without prejudice. Anonymity and confidentiality were observed as the names of either the participants or institution were never used. A USD10 reimbursement was provided to each participant per each visit to cover transport costs.

### Findings

#### Biographic information

The study participants consisted of 20 females aged between 25 and 45 years, and self-identified as sex workers. All participants had no other form of employment apart from sex work.

### Factors affecting access to HIV care services

#### Perception of the quality of care Positive Perceptions

The services were perceived to be comprehensive and the nurses to be caring. An example is reflected by the following extract: “*I also get additional services such as TB screening, cervical cancer screening and viral load monitoring. If I am going for counselling, they check if the viral load was done. The nurses also have a caring attitude*.” (42 years, 4 years on ART, private clinic)

#### Negative perceptions

On the other hand, nurses were perceived to be non-caring and clients were afraid to approach or ask for any clarification as displayed by these extracts: “*I don't ask, because the nurses have a non-caring attitude and they will tell you there are many people outside to be attended to, so that opportunity is missing*.” (40 years, 29 months on ART, state owned facility)

“*I had an experience where I had misplaced my O.I (Opportunistic Infections) client care card, went to the clinic with my old card, and also knew my file number. Explained my situation but I was in an indirect way punished, served last. When I had come at 7am and only to be attended to at 4pm*.” (42years, 5years on ART, state owned facility)

#### Delays in service provision

The majority of clients indicated that the services were slow: “*The services are very slow. The clinic opens at 8am, the nurses start serving the clients at 10 or 11 am, after having arrived at the clinic at 3am*.” (37 years, 35 months on ART, state owned facility)

Also, the clients felt that the set review dates were not convenient for them because of their job: “*...the problem is that at times the review date that I am given coincides with the time/month-end I would be busy. When these dates are given, nurses expect us to keep the appointment, which becomes a challenge*.” (33 years, 30 months on ART, state owned facility)

Because of their work, these clients preferred a booking system and expressed it thus: “*The problem is that at the clinic we are not afforded appropriate time to get our medicines supplies, at times I do not sleep at home, would be in the streets looking for money, hence I prefer a booking system*.” (43 years, 3 years on ART, state owned facility)

However, the situation was different at a private setting as shown in the extract: “*Since I go to the facility on a booked appointment, I have never experienced any delays, I am always served on time*.” (35 years, 2years on ART, private clinic)

### HIV Related Factors

#### Counselling Content

Counselling content was comprehensive as displayed by the following extract: “*Counselling included adherence to ART, disclosure, safer sex in relation to my profession and the importance of keeping review dates*.” (25 years, 2 years on ART, private clinic)

Whilst counselling was perceived to be comprehensive, focusing on specific HIV care related issues it was not individualised as shown by the citation below: “*The nurses do not know that I am a sex worker because they have never asked me what I do for a living. Counselling dwelt on general issues such as taking of ARVs, viral load monitoring and disclosur*e” (37years, 5 years on ART, state owned facility)

Care was generalised as shown in the following extract: “When we get to the clinic, we get the cards to the clerk for stamping. Thereafter, we go and queue for tablets, “*just hear next, next, use the next system*”. *The nurses do not even talk to you, in most instances they will be charting to each other*”. (34 years, 3 years on ART, state owned facility)

### Stigma and Discrimination

The majority of clients never disclosed to the health workers that they did sex work, however those that were brave enough to disclose, felt that the service providers displayed discriminatory behaviour: “ *After asking me about what I do for a living, I told them I was a sex worker and they started calling me names*.'’ They called each other and made remarks such as “*Why do sex work at this age? Is there nothing else that you can do besides sex work?*” (35 years, 32 months on ART, state owned facility)

On a related note, one participant said: “*Instead of them getting to understand what made me get into sex work, they made concluding remarks without understanding my background. Furthermore, they were asking personal & sensitive questions such as how do you charge?*” (32years, 37 months on ART, state owned facility)

The findings also show that elements of stigmatization interfered with retention in care, pushing some study participants to self-transfer. The statement below shows how the client expressed herself: “*I left ..., without telling them that I am transferring to this facility, but they have never followed up to check on me since the past two years. At this facility I have not experienced any form of stigma. Besides, they have a booking system which works out convenient for me*” (40 years, 4 years on ART, private clinic)

### Disclosure

Due to the fact that sex work is criminalised in Zimbabwe, the majority of participants in this study found it unnecessary to disclose to the service providers that in actual fact they earn a living by doing sex work: “*I heard others indicating they went for STI treatment and nurses disclosed information to other people or got embarrassed in-front of other clients. I found it convenient for me not to disclose that I am a sex worker. Even then they have never asked how I earn a living*.” (29 years, 3 years on ART, state owned facility)

However, another client had a different experience as shown thus: “*The nurse made an indication of the importance of talking about myself, so that she could understand me for better care. I disclosed that I was a sex worker on the first visit at the ART facility. I was treated just like any other client and did not feel discriminated*”. (35 years, 2years on ART, private clinic)

### Social Support

The participants in this study indicated that they got support from their peers and family members as depicted below:

“*I got support from my friends/colleagues for example reminder about time of taking the medication and accompaniment to the clinic. I was also supported by peer counsellors at this facility*” (39 years, 4 years on ART, private clinic)

“*My mother has been my pillar of strength. During the first days she would go with me to the clinic*.” (28 years, 18 months on ART, state owned facility)

### Socio-economic Factors

#### Travelling Costs

Distance and related transport costs became a factor because some sex workers preferred to access HIV care and treatment services in facilities away from their places of residence or work. Prior to this there were no transport costs as services were accessed locally. They felt that this would give them some kind of anonymity and not compromise their work: “*Because I did not want to meet my neighbours or the nurses from the nearby clinic, I preferred to get my ARVs from another clinic*.” (35 years, 3 years on ART, state owned facility)

The participants acknowledged the additional travel costs, but still felt the advantages outweighed the disadvantages. The extract below shows that: “*True, there is a cost due to distance to the other clinic, but it is better than having my identity exposed and leading to possible loss of income from my clients. However, I have to wake up at 3am on the day of review*.” (30 years, 39 months on ART, state owned facility)

### Loss of earning when not selling

The majority of sex workers felt that they were inconvenienced and had loss of earnings because of spending a lot of time in the clinic during review days: “*... but my challenge was that I used to stay at X Suburb but getting my supplies at Y Suburb clinic. I would wake up at 4am, only to be served at 12 pm. I wish I could get a place where I am served on time*.” (36 years, 4 years on ART, state owned facility)

## Discussion

One of the key study findings was that care and counselling at state owned facilities were generalised. There were also incidences of stigma and discrimination as well as delays in service provision. The situation was, however, different at private facilities.

This study adds to the body of knowledge by other researchers in the region^20^,^21^. The findings of this study will add to the corpus of knowledge and assist in programming HIV services for this group and assist in policy formulation for differentiated care services for key populations. The findings suggest that the health care system can either be a barrier or a facilitator to accessing HIV care and treatment services. Good quality health services found expression in the form of polite expressions and caring nurses as well as follow up structures. Related studies on the comprehensiveness of the HIV care package in Rwanda and Nigeria also concur with these findings^22^,^23^. Conversely, barriers identified were negative staff attitudes, breaches of confidentiality, hostility and humiliating comments, stigma and discrimination as was found in other studies^20^,^24^,^25^,^26^. The aforementioned positive and negative experiences found in this study are also documented in other studies^27^,^28^. Negative attitudes by health service providers lead to non-disclosure by clients of their profession (sex work). There is need for in-service training of healthcare workers that sensitizes them to offer non-discriminatory services and eliminating stigma against female sex workers. There is sufficient evidence of the effectiveness of this approach as was demonstrated by studies in South Africa^29^,^30^.

This and other studies, demonstrate the key role of socio-economic factors in accessing HIV care services^29^. Proximity to health facilities was seen as an advantage by some as it resulted in cost cutting. However, another group preferred to defy the resultant transport costs and longer distances since they preferred the anonymity given by the distance from their work sites or residential places. These findings concur with those by other researchers^27^,^28^.

Negative attitudes by health service providers lead to non-disclosure by clients of their profession (sex work). The findings on HIV related services are equally illuminating. While the experiences are not homogeneous, they corroborate studies elsewhere, about the attitude of health workers towards female sex workers and other key population groups^5^,^27^,^31^,^32^,^33^. These groups experienced negative and uncaring attitudes from health workers when they visited the health care facilities. However, this is by no means a homogenous experience as some reported a positive, caring, and professional attitude, exclusively in private clinics. This could be attributed to the fact that the private clinic offers HIV care and treatment services that are specifically dedicated to the key populations. To reduce prejudice against sex workers and foster an enabling environment for the provision of HIV care services for this and other key population groups, there is need for sensitization and training of health-care cadres to fully understand female sex workers and their unique service delivery needs^30^. Socio-cultural attitudes towards sex workers are not unique to the health care settings, but are prevalent in societies in general, as demonstrated by studies in the region 27,28.

Studies in the region have demonstrated that making health services accessible and acceptable for sex workers was a critical enabler^29^,^34^. In this study, HIV care services were made inaccessible due to long waiting times in public sector facilities. The delays and long waiting times are an inconvenience to this group of people as they experienced loss of earnings as other study findings show^20^,^21^,^27^,^28^,^35^. Additionally, some participants in this study arrived at the public sector facilities as early as 3am to have a better chance of being served. The recommendation would be that health care facilities operate flexible opening hours that are accessible and convenient for FSW. This concurs with the findings of a study in Uganda^36^. However, the experiences were different in the private clinic since a booking system was being utilised. Studies in Ethiopia and Kenya established that the use of an appointment system significantly improved patient waiting time in HIV care clinics^37^,^38^.

Health access navigation and social support from peers and family as found in this study, is an important component in linkage to HIV care as was demonstrated in such studies as in Uganda, Zambia, Ethiopia and elsewhere^27^,^39^,^40^,^41^,^42^. There is need to develop strategies that build on peer social support^35^,^43^,^44^. The use of peers(sex workers) colleagues in the same business was pivotal particularly at the private clinic, as other studies demonstrate^27^,^45^.

According to the WHO, differentiated service is a client-centred approach that simplifies and adapts HIV services across the cascade to reflect the preferences and expectations of various groups of people living with HIV^13^,^40^,^41^. The findings of this study, with reference to the public sector health facilities were different from what is advocated for, since counselling and care were generalised. Therefore, clinical competency training for health workers would be essential to enhance their ability to obtain relevant health history and form the basis of counselling. This is borne out by the Ethiopian experience in the Mulu MARPs (most atrisk populations to HIV infection) sex worker project which demonstrated that training and sensitising health care workers in key population friendliness and clinical competence improved linkage and retention in care^46^. Consistent with these findings are studies by Duby et al, Poteat et al and Geibel et al 30,47,48. In addition to this, the researchers recommend more separate sex worker friendly clinics that provide integrated HIV care services that have different and flexible operating times so as to decrease waiting times among other things^33^,^49^,^50^. As a mechanism of enhancing access to HIV prevention, care and treatment services, it is recommended that multiple strategies such as the use of mobile outreaches, brothel-based clinics and moonlighting for provision of services for sex workers be adopted. This is supported by experiences from studies elsewhere^26^,^49^,^50^,^51^,^52^. This will facilitate provision of differentiated client centred services. Finally, it would be critical for health care providers in private sex worker clinics to share best practices and lessons learnt with their public sector counterparts.

One of the limitations of the study was that it was confined to only one city and the findings may not necessarily apply to different settings. Perhaps similar studies in other cities in the country may be carried out. Another limitation was that participants below the age of 18 years were not represented in the sample making generalisability difficult.

## Conclusion

These findings underscore the need to design interventions that require multi-pronged approach to increase linkages to HIV care services and retention in care for sex workers. From the study, it is clear that accessing HIV care services for female sex workers is dependent on quality friendly services that are accessible, client centred and free from stigma. There is need for service providers to be empowered about the dynamics of the key population groups (sex workers) and their professional work so as to meet the health needs in a comprehensive manner.

## Figures and Tables

**Table 1:** Summary of superordinate themes, themes and sub-themes

Superordinate theme	Themes	Sub-themes
**Access to HIV** **Care Services**	Health Care System factors	Perception about quality of care
		Positive Perception
		Negative attitude of service providers
		Delays in service provision
	HIV Related Factors	Counselling content
		Stigma and discrimination
		Disclosure
		Social support
	Socioeconomic factors	Travelling costs
		Loss of time when not selling
